# Evolutionary history of the *mariner* element *galluhop* in avian genomes

**DOI:** 10.1186/s13100-017-0094-z

**Published:** 2017-08-14

**Authors:** Natasha Avila Bertocchi, Fabiano Pimentel Torres, Analía del Valle Garnero, Ricardo José Gunski, Gabriel Luz Wallau

**Affiliations:** 1 Programa de Pós-graduação em Ciências Biológicas, Universidade Federal do Pampa (Unipampa), São Gabriel, Rio Grande do sul 97300-000 Brazil; 2 Laboratório de Diversidade Genética Animal, Universidade Federal do Pampa (Unipampa), São Gabriel, Rio Grande do sul 97300-000 Brazil; 3 Departamento de Entomologia, Instituto Aggeu Magalhães – FIOCRUZ-CPqAM, Recife, Pernambuco Brazil

**Keywords:** *Galluhop*, *Mariner*, Avian genome, Horizontal transfer, MITEs, Genomic parasites

## Abstract

**Background:**

Transposable elements (TEs) are highly abundant genomic parasites in eukaryote genomes. Although several genomes have been screened for TEs, so far very limited information is available regarding avian TEs and their evolutionary histories. Taking advantage of the rich genomic data available for birds, we characterized the evolutionary history of the *galluhop* element, originally described in *Gallus gallus*, through the use of several bioinformatic analyses.

**Results:**

*galluhop* homologous sequences were found in 6 of 72 genomes analyzed: 5 species of Galliformes (*Gallus gallus*, *Meleagris gallopavo*, *Coturnix japonica*, *Colinus virginianus*, *Lyrurus tetrix*) and one Buceritiformes (*Buceros rhinoceros*). The copy number ranged from 5 to 10,158, in the genomes of *C. japonica* and *G. gallus* respectively. All 6 species possessed short elements, suggesting the presence of Miniature Inverted repeats Transposable Elements (MITEs), which underwent an ancient massive amplification in the *G. gallus* and *M. gallopavo* genomes. Only 4 species showed potential MITE full-length partners, although no potential coding copies were detected. Phylogenetic analysis of reconstructed coding sequences showed that *galluhop* homolog sequences form a new *mariner* subfamily, which we termed *Gallus*. Inter-species and intragenomic *galluhop* distance analyses indicated a high identity between the consensus of *B. rhinoceros* and the other 5 related species, and different emergence ages of the element between the Galliformes species and *B. rhinocerus*, suggesting that horizontal transfer took place from Galliformes to a Buceritiformes ancestor, probably through an intermediate species.

**Conclusions:**

Overall, our results showed that *mariner* elements have amplified to high copy numbers in some avian species, and that this transposition burst probably occurred in the common ancestor of *G. gallus* and *M. gallopavo.* In addition, although no coding sequences could be found currently, they probably existed, allowing an ancient massive MITE amplification in these 2 species. The other 4 species also have MITEs, suggesting that this new *mariner* family is prone to give rise to such non-autonomous derivatives. Last, our results suggest that a horizontal transfer event of a *galluhop* element occurred between Galliformes and Buceritiformes.

**Electronic supplementary material:**

The online version of this article (doi:10.1186/s13100-017-0094-z) contains supplementary material, which is available to authorized users.

## Background

Transposable elements (TEs) are widely distributed and abundant component of many eukaryotic genomes. TEs can be classified in two main classes, based on their transposition mechanism: Class I (moves through an RNA intermediate) and Class II (through a DNA intermediate) [1–3]. Successful proliferation of TEs in genomes is linked to their replicative and mobile capacity within the host genome and also between genomes [4, 5]. On the other hand, most of the time this mobility is neutral or deleterious to the host organism. New TE insertions in gene-coding regions or in upstream/downstream positions can have a drastic impact on flanking genes [6]. These highly similar and repetitive sequences throughout the genome also provide a substrate for ectopic recombination events that can lead to chromosomal inversions and deletions [7, 8]. However, an increasing body of evidence is showing that insertions of new TEs introduce variability and can sometime be adaptive for the host genome [9, 10].

TEs are an integral part of host genomes and hence are vertically transmitted to descendants through the male and female germ line DNA, and from ancestral to extant species in the course of evolution [11]. However, compelling evidence in a wide variety of taxa has increasingly revealed that Horizontal Transfer (HT), the exchange of genetic material between isolated sexual species, is an effective way in which TEs invade new genomes and colonize other species [11, 12]. Currently, around 2853 Horizontal Transposon Transfer (HTT) events have been reported [13]. The *mariner* family of Class II DNA transposons has the highest number of HTT cases reported (52) [13, 14]. Such events have been characterized in a wide variety of taxa, including insects and mammals [14–16]. In birds, considering all TE families of Class I and II, only seven HTT events have been reported so far: two retrotransposons (AviRTE), which took place between several bird species ancestors and human pathogenic nematodes [17].

Non-autonomous elements can emerge at any step of the TE “life cycle” through deletion or internal region degeneration, yet retain their transposition capacity in the presence of autonomous or coding copies**.** Internally deleted non-autonomous elements originating from Class II transposons are known as Miniature Inverted-repeat Transposable Elements (MITEs) [2]. These elements possess deletions or a degenerated coding region, but preserved Terminal Inverted Repeats (TIRs) which can be recognized by functional transposases [18, 19]. MITEs have been associated with several Class II superfamilies such as hAT, *P* and Tc1/mariner [20–22]. Usually MITEs reach higher copy numbers than their autonomous counterparts, a form of parasitism that may lead to the extinction of the entire TE family in the long term [23].

Although TEs are currently recognized as major players in genome evolution, in some taxa such as birds, knowledge of TEs is limited [24, 25]. One of the reasons for this gap has been the scarcity of available genome sequences, but since 2014, more than 70 draft whole genome sequences have become available [26]. Among the few studies focusing on TEs in bird genomes, a reduction in repetitive DNA was detected in sauropsids, perhaps due to the purifying selection pressure acting on metabolism optimization [25, 27, 28]. In particular, Class II TEs, which are abundant in other eukaryotic species, appear to show limited diversity in the few avian genomes studied so far: the chicken *Gallus gallus* and the wild turkey *Meleagris gallopavo* [24, 29].

Elements from the *Tc1-mariner* superfamily generally are 1.3 kb long, and contain TIRs of approximately 28 bp and a unique ORF (Open Reading Frame) which codes for a transposase [30, 31]. Because of the great diversity of the *mariner* family, these elements were classified in subfamilies based on phylogenetic analyses. The classification proposed by Rou﻿ault et al﻿ [32] includes 12 subfamilies (*mauritiana, cecropia, rosa, mellifera, lineata, capitata, irritans, briggsae*, *elegans, Atlantis* and *CRI*). Among the Class II TEs found in avian genomes, a *mariner*-like element termed *galluhop* was previously characterized [29, 33], but up to now no other study has focused on understanding its evolution in other avian species.

Here, we aimed to characterize the evolutionary history of *galluhop* homolog sequences found in available avian genomes. Our results showed that *galluhop*-like sequences compose a new *mariner* subfamily, which was exchanged between two bird taxa through horizontal transfer, probably mediated by an intermediate species.

## Methods

### Bioinformatic workflow

#### Genome search for *galluhop* homologs

The nucleotide sequence from the *galluhop* consensus described by Wicker et al. [33] was obtained from the Repbase database [33–35]. 72 avian genomes were available as of May 2016 (Additional file 1: Table S1). BLASTn searches were performed using the *galluhop* consensus sequence from Repbase, using default parameters. Only blast results with an E-value lower than e^−10^ were analyzed further. *In house* python scripts were used to retrieve all sequences and 200 base pairs of flanking sequences from each copy.

Sequence alignments of all copies plus flanking sequences from each species were performed with MAFFT v.7 [36] (Additional file 2: Figure S1).

#### Functional characterization

The resulting alignments were manually inspected and corrected in order to precisely identify TIRs and target site duplications. TIRs conservation was determined visually, using Weblogo [37]. After identification and definition of element copy boundaries, all copies were characterized by the presence of ORFs, using the OrfFinder script implemented in UGENE [38] and the script implemented in Emboss gertof (http://emboss.sourceforge.net/apps/cvs/emboss/apps/getorf.html) with the following parameters: -minsize 900 -find 1 -methionine Y. Copies were classified as i) possessing a predicted coding protein = > than 300 aa and conserved TIRs as potential autonomous copies; ii) possessing a potential coding protein <= than 300 aa and conserved TIRs as potential non-autonomous copies; iii) copies with a missing TIR but with ORFs = > than 300 aa as potential coding copies; and iv) elements with a missing TIR and ORFs <= 300 aa as partial elements (Additional file 2: Figure S1).

#### Nucleotide distance and phylogenetic analysis

In order to estimate the interspecies distance of TEs, we reconstructed the majority consensus ancestor element with all copies found per genome, using UGENE [38]. The Kimura 2 parameter (K2P) distance between all copies and their corresponding consensus sequence was estimated with the distmat script from the Emboss package (http://emboss.sourceforge.net/apps/release/6.6/emboss/apps/distmat.html) and histogram distribution plotted with ggplot2 [39] in the R environment [40]. Dating between *galluhop* consensus elements and copies within each genomes was performed according to the eq. T = k/2r [41]. T represents the divergence time between TEs, k is the divergence value between the TE consensus and copies, and r is the mean evolutionary rate for bird genomes [41]. We used species-specific evolutionary rates when available, or the closest relative rates: *Gallus gallus* 1.9 × 10^−3^, *Meleagris gallopavo* 2.0 × 10^−3^, *Buceros rhinoceros* 2.3 × 10^−3^, *Lyrurus tetrix* 1.9 × 10^−3^, and *C. virginianus* 1.9 × 10^−3^ [42]).

We also obtained the coding regions of 50 single-copy orthologous genes between the *B. rhinoceros* and *L. tetrix* genomes, and estimated the K2P distance in order to compare with the TE K2P distance. The OrthoDB database [43] was used to search single-copy orthologous genes found in all 52 available avian genomes analyzed in this database version. Due to the lack of data for *L. tetrix* in the database, we used the mRNA accession number of *B. rhinoceros* as the blastn query against the *L. tetrix* genome in order to obtain the gene sequence used for the latter.

Alignments of reconstructed *galluhop* coding region (almost complete ORF and partial for those composed only for MITEs) from all 6 species that possessed *galluhop* homolog sequences were performed, using a previously published transposase alignment covering most of the *mariner* subfamilies [41], using MAFFT v.7 [36].

Phylogenetic reconstruction was performed by maximum likelihood, using PHYML [44], and branch support was evaluated by SH-like support [45].

## Results and discussion

### *galluhop* homologs in bird genomes

Six of the 72 avian genomes analyzed harbored *galluhop*-like sequences (Table 1). Five of these are from species of the order Galliformes that diverged from each other at least 46 Mya (CI: 37 – 55 Mya – [46]): *Colinus virginianus, Coturnix japonica, Lyrurus tetrix, Gallus gallus* and *Meleagris gallopavo.* We also identified *galluhop-*related sequences in *Buceros rhinoceros* of the order Bucerotiformes, which diverged from Galliformes 98 Mya (CI: 92.1–104.0 Mya – [46]). Consensus of *gallohop per* species can be found in Additional file 3. Table 1 shows the Kimura 2 parameter distance between the consensus element from each species.

**Table 1:** Kimura 2 parameter distance between each *galluhop* consensus sequence

*M. gallopavo*	*L. tetrix*	*G. gallus*	*C. japonica*	*C. virginianus*	*B. rhinocerus*	Species
						*B. rhinocerus*
					0.0921	*C. virginianus*
				0.1654	0.1843	*C. japonica*
			0.1691	0.0519	0.0755	*G. gallus*
		0.0416	0.141	0.0519	0.0571	*L. tetrix*
	0.0382	0.0225	0.166	0.0535	0.0797	*M. gallopavo*

**Fig. 1:**
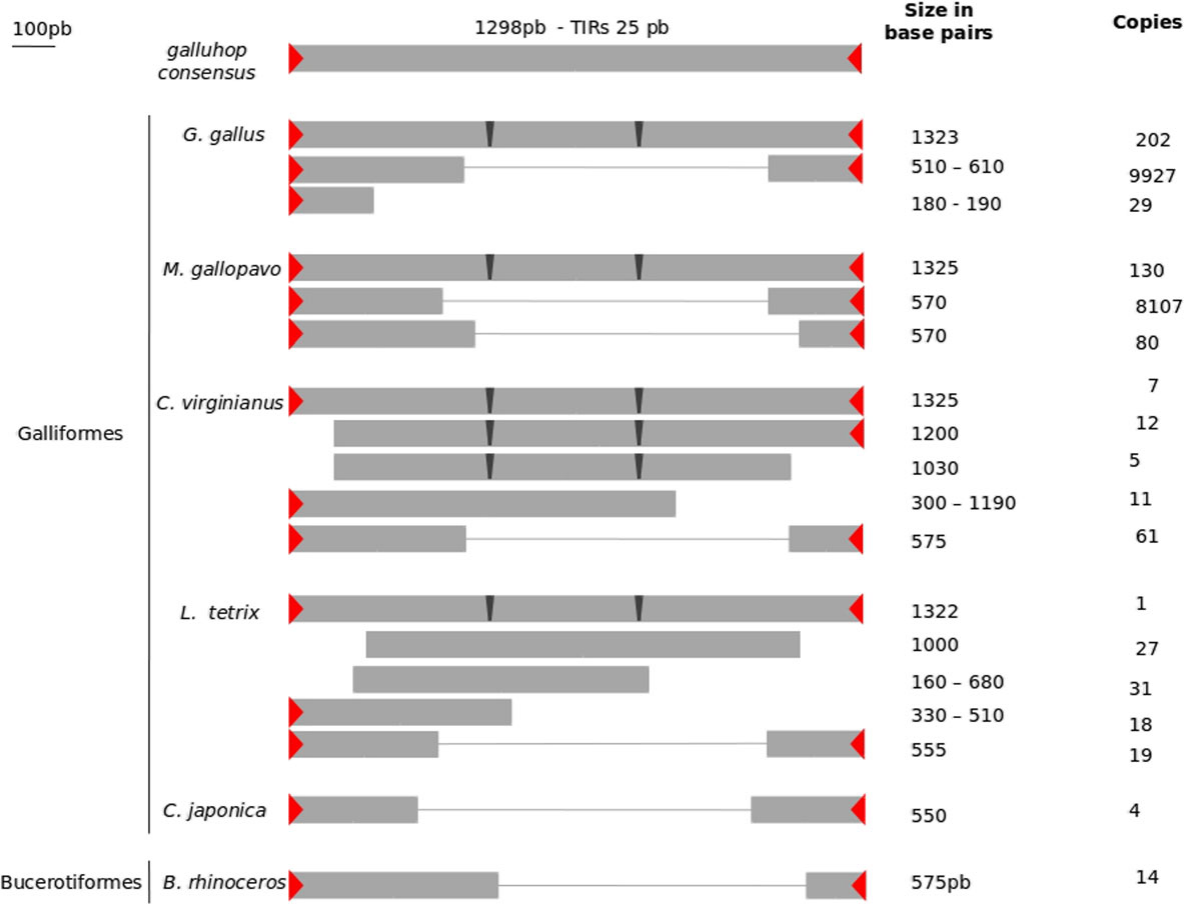
Schematic representation of the reconstructed *galluhop* copies compared to the *galluhop* consensus. Regions of terminal inverted repeats shown in *red*, transposase coding region in *light gray*, and insertion region in *dark gray*. Order Galliformes: four genomes (*G. gallus, M. gallopavo, C. virginianus* and *L. tetrix*) showed potential complete partners although there are no potential coding copies, and *C. japonica* showed short elements. Order Bucerotiformes: *B. rhinoceros* showed only short elements

These elements reached a high copy number in both the *G. gallus* and *M. gallopavo* genomes, 10,158 and 8317 respectively. The remaining 4 species showed lower copy numbers, from 5 to 96 copies (Table 2). No potential autonomous or coding copies were found (Table 2). Four of 5 Galliformes species (*G. gallus, M. gallopavo, C. viginianus* and *L. tetrix*) showed elements with a similar size to the reference *galluhop* element deposited in Repbase (around 1300 bp; Table 1), although most of them showed two 12-bp insertions that prevented any transposase from being fully encoded (Fig. 1). The remaining species of Galliformes, *C. japonica*, showed only 5 short elements of 550 bp and conserved TIRs resembling MITEs in the assembly version analyzed. Last, *B. rhinocerus* showed 14 copies of 575 bp but with conserved TIRs and subterminal regions of the elements (Fig. 1 and Additional file 4 Figure S2). Most of the *galluhop*-like sequences found showed both imperfect TIRs (Additional file 4: Figure S2) and target site duplication (TSD) TA characteristic of *mariner* elements (Additional file 5: Figure S3).

**Table 2:** Avian genomes with *galluhop* and characteristics of copies

Partial elements (160–1200 bp)^b^	Non-autonomous elements (~500–600 bp)	Full-length elements (~1200–1300 bp)	ORFs^a^	No. of copies	Assembly size (Mb)	Species	Order
29	9927	202	N	10,158	1046.93	*G. gallus*	Galliformes
0	8187	130	N	8317	1061.82	*M. gallopavo*	Galliformes
28	61	7	N	96	1171.86	*C. virginianus*	Galliformes
76	19	1	N	96	657.025^c^	*L. tetrix*	Galliformes
0	4	0	N	4	531.96^c^	*C. japônica*	Galliformes
0	14	0	N	14	1065.78	*B. rhinocerus*	Buceritiformes

^a^No ORFs were found in the analyzed elements

^b^Partial elements are copies with a missing TIR and ORFs <= 300 aa

^c^ *L. tetrix* and *C. japonica* genomes have a smaller assembly size than most avian genomes, since they are only partially assembled. A new assembly version of the *C. japonica* genome is available, with a higher assembly size of 927.657 Mb – GCA_000511605.2, but it was not used in our study since it was released after we conducted all analyses in the previous assembly version

**Fig. 2:**
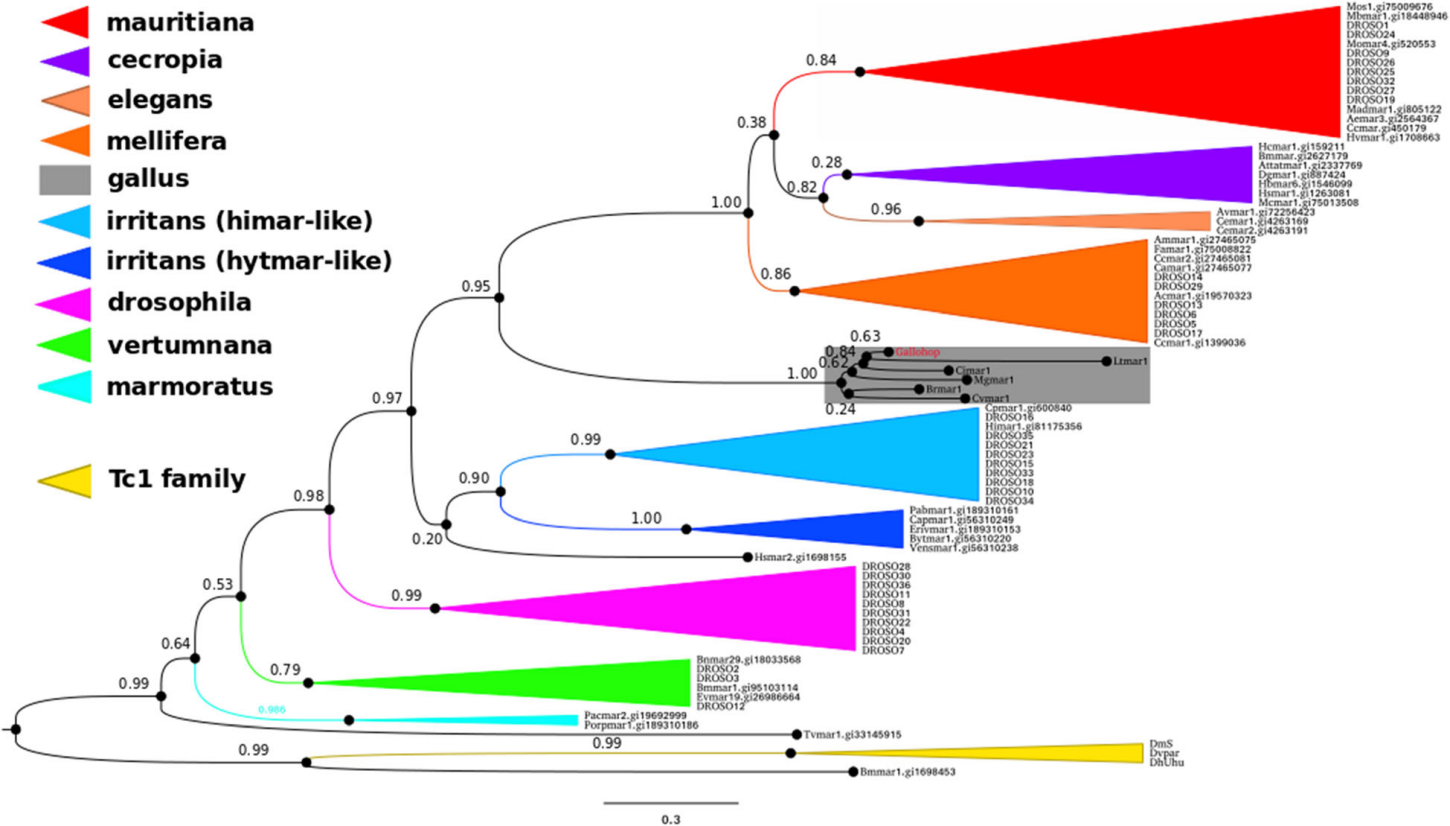
Phylogeny of *mariner*-like transposases*.* Phylogeny of *mariner*-like transposases, by maximum likelihood using PHYML (Guindon and Gascuel 2003). *Clade colors* denote the different subfamilies of the *mariner* family, indicated to the *left* of the tree. In *gray*: the new subfamily, *Gallus*

Phylogenetic analysis using all *galluhop*-like consensus sequences and several sequences from the *mariner* subfamilies indicates that *galluhop*-like elements compose a new *mariner* subfamily, which we termed *Gallus* (Fig. 2)*.* TEs from the *Gallus* family emerged in the ancestor of the order Galliformes (around 55–65 Mya) [46], increasing its copy number, particularly in the *G. gallus* and *M. gallopavo* genomes*.* Only non-autonomous copies of the *Gallus* subfamily were found possessing several mutations, multiple stop codons and changes in the element reading frame (Fig. 1). We also found a large number of short non-autonomous elements (around 500–600 bp) with preserved 5′ and 3′ regions of the element, including TIRs (Fig. 1 and Additional file 4: Figure S2), but with a large deletion compared with the full-length consensus element (Fig. 1). These shorter elements showed all the characteristics of MITEs [19] and amplified successfully in *G. gallus* and *M. gallopavo,* composing the large majority of *galluhop* copies found in these genomes (97.7% in *G. gallus* and 98.4% in *M. gallopavo* genomes). *C. virginianus* and *L. tetrix* also showed amplification of MITEs on a smaller scale, and *C. japonica* and *B. rhinocerus* possessed only MITEs elements and no trace of their possible autonomous counterparts (Fig. 1). Taken together, these findings suggest that MITEs originated independently in this new *mariner* subfamily, which probably affected the fate of these elements leading to the extinction of the TE family in all avian genomes studied. This view is in agreement with the hypothesis that the emergence of superparasites such as MITEs can lead TE families/subfamilies to decay and disappear over time [19, 47].

### *galluhop* intra- and interspecies evolution

**Fig. 3:**
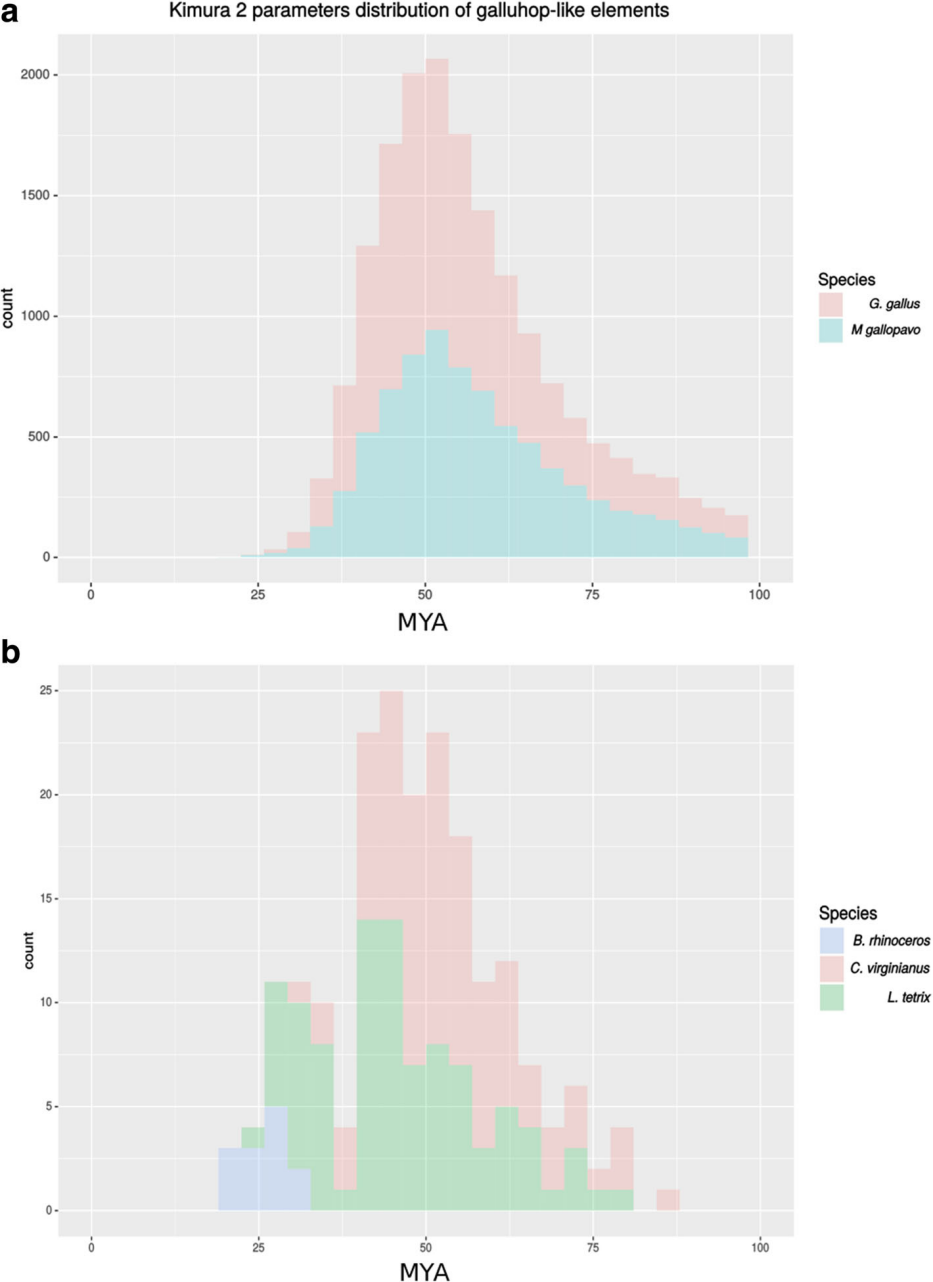
Amplification dynamics of elements within each genome in million of years. **a** Intragenomic dating of copies found in *G. gallus* and *M. gallopavo*. **b** Intragenomic dating of copies found in *B. rhinocerus*, *C. virginianus* and *L. tetrix*

The intragenomic divergence between each *galluhop* copy and its corresponding ancestor consensus sequence was calculated in order to infer the time frame of TE arrival and their amplification dynamics in each genome, except for *C. japonica,* due to the low copy number in this genome (Table 2). Making use of species-specific or the closest-relative evolution rates, we could estimate this dynamic in millions of years ago (MYA). As seen in Fig. 3a and b, the species of Galliformes showed a wide distribution of element ages, with a single peak ocurring in *G. gallus* and *M. gallopavo* between 100 and 25 MYA (Fig. 3a), and two peaks in *C. virginianus* and *L. tetrix* at around 87.5 and 37.5 MYA and 37.5 and 18.75 MYA (Fig. 3b), suggesting that these elements are ancient parasites of galliformes genomes and increased in copy number through single or double amplification waves. However, the only buceritiformes species bearing *galluhop* elements, *B. rhinocerus*, showed a much younger element distribution ranging from 31.5 and 18.75 MYA, suggesting a single, more recent, amplification wave (Fig. 3b).

These differing amplification age distributions could be interpreted as due to the differing evolutionary rates between the species analyzed, and not due to different emergence and amplification dates of the TEs. The *B. rhinocerus* genome has the highest evolutionary rates of the species analyzed here, suggesting that if this bias is real, we would expect to observe lower than expected element ages in this species biasing our analysis. In order to evaluate if lower evolutionary rates could significantly change the estimates for *B. rhinocerus* elements, we used the evolutionary rate for water birds (1.6 × 10^−3^) [42], which is one of the lowest estimates for birds, to estimate the *B. rhinocerus galluhop* invasion. Even so, we obtained ages for *B. rhinocerus* elements between 31.2 and 43.7 MYA, which is still much younger than all estimates for the origin of *galluhop* in galliform genomes, suporting the hypothesis that *galluhop* emerged in *B. rhinocerus* more recently than in Galliformes.

Younger element ages in *B rhinocerus,* a species from the Neoaves, order Buceritiformes, combined with a patchy distribution of *galluhop* in the avian tree, found in only 5 additional galliform species (*C. virginianus, C. japonica, L. tetrix, G. gallus* and *M. gallopavo*) which diverged from *B. rhinocerus* around 85–98 MYA [46], suggests that probably a horizontal transfer event took place directly between the common ancestor of these taxa or through an intermediate species.

**Fig. 4:**
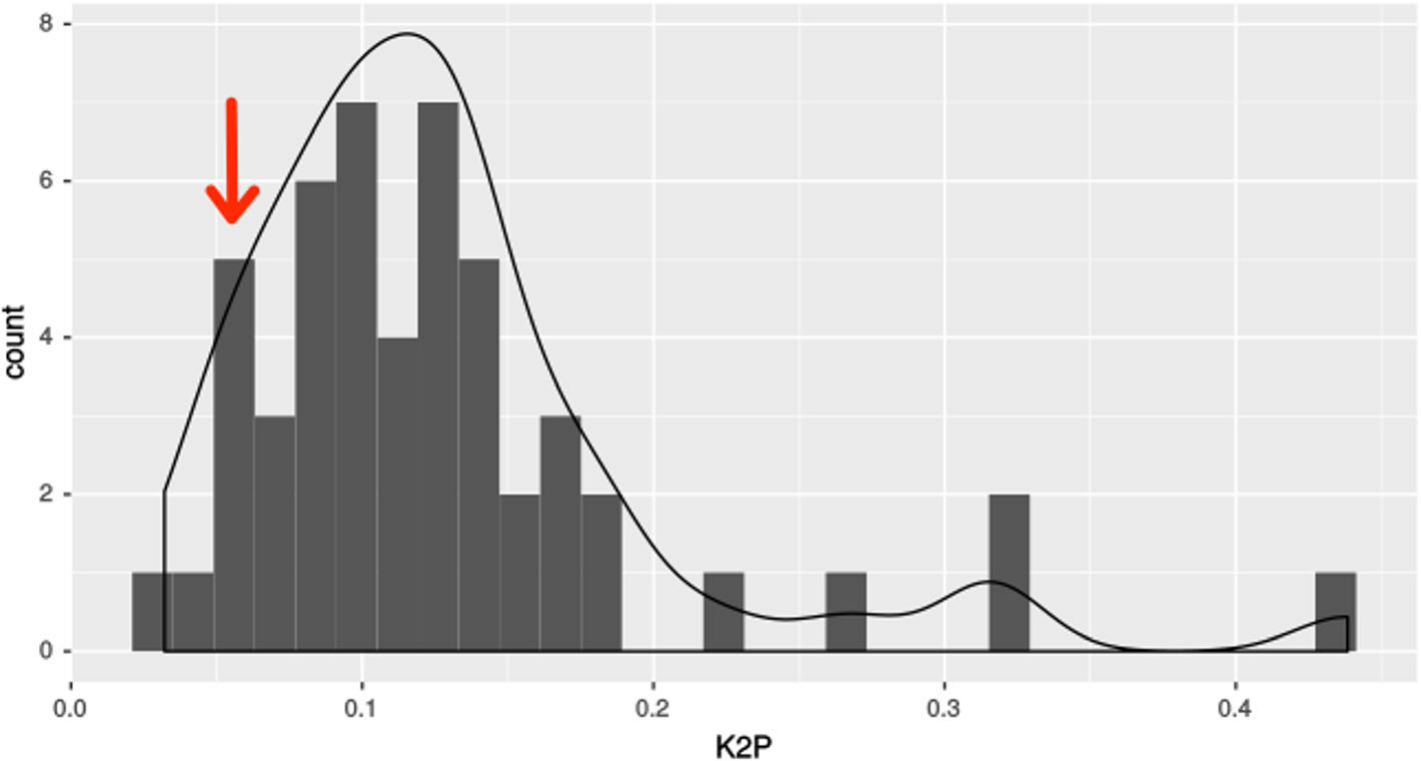
Density plot of Kimura 2 parameter distance between *B. rhinocerus* and *L. tetrix*. K2P distance of 50 single-copy orthologous genes (*gray shading*) and consensus TEs (*red arrow*)

In order to gain additional insights about possible donor and receptor species, we first evaluated the evolutionary distance of species-specific TEs consensus sequences. Among all galliform consensus sequences, the distance at the nucleotide level varied from 0.0382 to 0.1654 (Table 1). The *B. rhinocerus* consensus showed a K2P distance of 0.0571 to 0.1843, being the lowest distance comparison with the *L. tetrix* consensus (Table 1). Second, we evaluated the evolutionary distance of the TEs consensus of *B. rhinocerus-L. tetrix* (K2P = 0.0571) with 50 single-copy host genes of each species. This reasoning is based on the following principle: a similar or higher TE-host gene distance is expected if TEs were evolving by vertical transfer, since they would have had the same time to accumulate mutations as host genes. On the other hand, a shorter TE distance compared with the host-gene distance is expected if a horizontal transfer took place. Figure 4 depicts an expected host-gene normally distributed K2P distance, with a tail for more divergent host genes. Most of the genes have an average K2P distance, and few genes have extreme values of low and high K2P, which can be explained by the negative and positive selection acting on them. The TE K2P distance (red arrow) is shorter than 92% of all host genes analyzed (46 genes) and falls in the extreme lower range of K2P values of host genes. Although one can think of this as an indication that the TE is evolving vertically since it has a similar distance as some host genes, TEs evolve neutrally, so we would expect to see vertically inherited TEs within the average host gene distance or at the opposite extreme of the distribution. Therefore, those results are in agreement with the HT hypothesis between *L. tetrix* and *B. rhinocerus* ancestor.

**Fig. 5:**
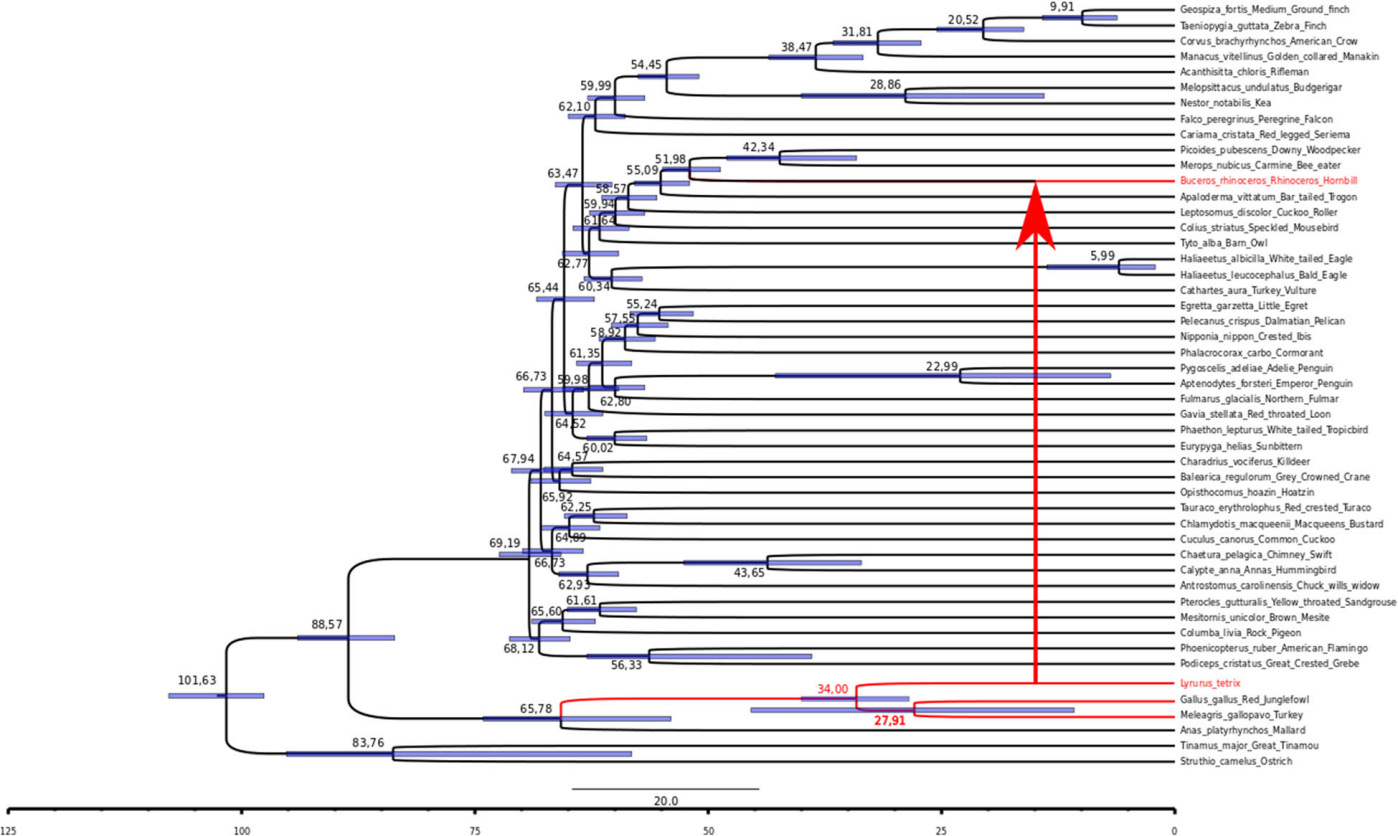
*Horizontal* Transfer hypothesis of *galluhop* elements*.* Chronogram TENT avian tree from Jarvis et al. [42] with the addition of probable *L. tetrix* positioning and split data following TimeTree data [44] *Red* branches denote the evolutionary hypothesis of the *Gallus* subfamily vertical evolution in the *Galliformes,* and *horizontal* transfer from *L. tetrix* to *B. rhinoceros* ancestors. *X bar* below the tree denotes the time in millions of years. Number close to nodes are the mean estimate of ancestors and blue bars are 95% credible interval as estimated by Jarvis et al [42]

One of the supporting lines of evidence which can shed light on time, direction and the presence of a possible intermediate species of an HTT event is the distribution of current and ancestors of the species involved and the element invasion dates. If host species have an overlapping distribution range and the estimates of element invasion are similar, then it is reasonable to suggest that HT occurred directly between them. Contrariwise, a non-overlapping range suggests that the HTT event occurred between the ancestors and different elements invasion ages through an intermediate species. *L. tetrix* and *B. rhinoceros* currently have distinct distribution ranges; the former is restricted to northern Eurasia, from the Swiss-Italian-French Alps to Scandinavia, Estonia and Russia; while the second occurs in Southeast Asia, including Borneo, Singapore, Malaysia and Thailand [48]. Fossils of other species of the genus *Lyrurus* and order Buceritiformes were found in Bulgaria and dated to the Miocene epoch (20.44 to 7.24 MYA) [49–51], although recent genome-wide paleogeographic inferences are few and limited, so that the ancestral distribution ranges of these two species cannot yet be defined with certainty [52]. Based on *galluhop-*like sequence ages, we observed that this element invaded *B. rhinocerus* ~ 31 MYA in the early Oligocene epoch of the Cenozoic era, while it arose in the *L. tetrix* genome around 75-82 MYA. Taken together, our data support an ancient HTT event between the ancestor of Galliformes and Buceritiformes or through an intermediate species; the latter is the most probable hypothesis, since different element ages were found (Fig. 5).

## Conclusions

The evolution of transposable elements usually shows complex patterns, such as patchy distributions within taxa, associated with a high similarity of TEs in host species that diverged long ago. The presence of such patterns can be explained by an exchange of TEs by these species or independent acquisitions from a third source, which characterizes a phenomenon known as HTT. HTT events have been reported throughout the eukaryote tree of life in recent years, and several of these events were reported for vertebrate species [13]. For instance, the SPIN transposon was found in more than 17 distantly related tetrapod species, including mammals as well as an African frog and a lizard, showing high similarity and patchy distribution [53, 54]. Despite these recent findings in vertebrates, only seven HTT events have been documented thus far, involving an avian clade and parasitic nematodes [17].

Here we evaluated the evolutionary history of the *mariner* element *galluhop* in Avian genomes. Our results shed new light on the phylogeny of the *mariner* family, describing a new subfamily termed *Gallus,* and highlights the successful amplification of MITEs of this subfamily in some avian genomes*.* We also report the first documented HTT event involving bird species. The analyses of the TE distribution, interspecies similarity and intragenomic element ages support the existence of the first HTT event between avian genomes.

## Additional files

Additional file 1: Table S1. GenBank access numbers for bird genomes. (XLSX 14 kb)

Additional file 2: Figure S1. Experimental design procedure showing steps of the analysis. Galluhop homologous sequences were found in 6 of 72 genomes analyzed. We analyzed the functional and structural characteristics and phylogenetic reconstruction of the putative transposases. (TIFF 4512 kb)

Additional file 3: Fasta consensus sequences. (FASTA 6 kb)

Additional file 4: Figure S2. Graphical representation of the conservation of terminal inverted repeats (TIRs). The TIRs 5′ and 3′ galluhop element in the six genomes generated with WebLogo [35]. Order Galliformes: *G. gallus* (A – A’),*M. gallopavo* (B – B′), *C. virginianus* (C – C′), *L. tetrix* (D – D’) and *C. japonica* (E – E’). Order Bucerotiformes: *B. rhinoceros* (F – F′). (TIFF 7747 kb)

Additional file 5: Figure S3. Graphical representation of the conservation of target site duplications (TSDs). The TSDs 5′ and 3′ galluhop element in the six genomes generated with WebLogo [35]. Order Galliformes: *G. gallus* (A – A’),*M. gallopavo* (B – B′), *C. virginianus* (C – C′), *L. tetrix* (D – D’) and *C. japonica* (E – E’). Order Bucerotiformes: *B. rhinoceros* (F– F′). (TIFF 4900 kb)

## Abbreviations

HTT: Horizontal transposon transfer
K2P: Kimura 2 parameters
MITE: Miniature inverted repeats transposable element
ORF: Open reading frame
TE: Transposable element
TIR: Terminal inverted repeat
TSD: Target site duplication
