# Supplementary figures and images for: Evolutionary history of the mariner element galluhop in avian genomes
Source: Mob DNA. 2017 Aug 14;8:11. doi: 10.1186/s13100-017-0094-z (PMC5556988; doi:10.1186/s13100-017-0094-z)

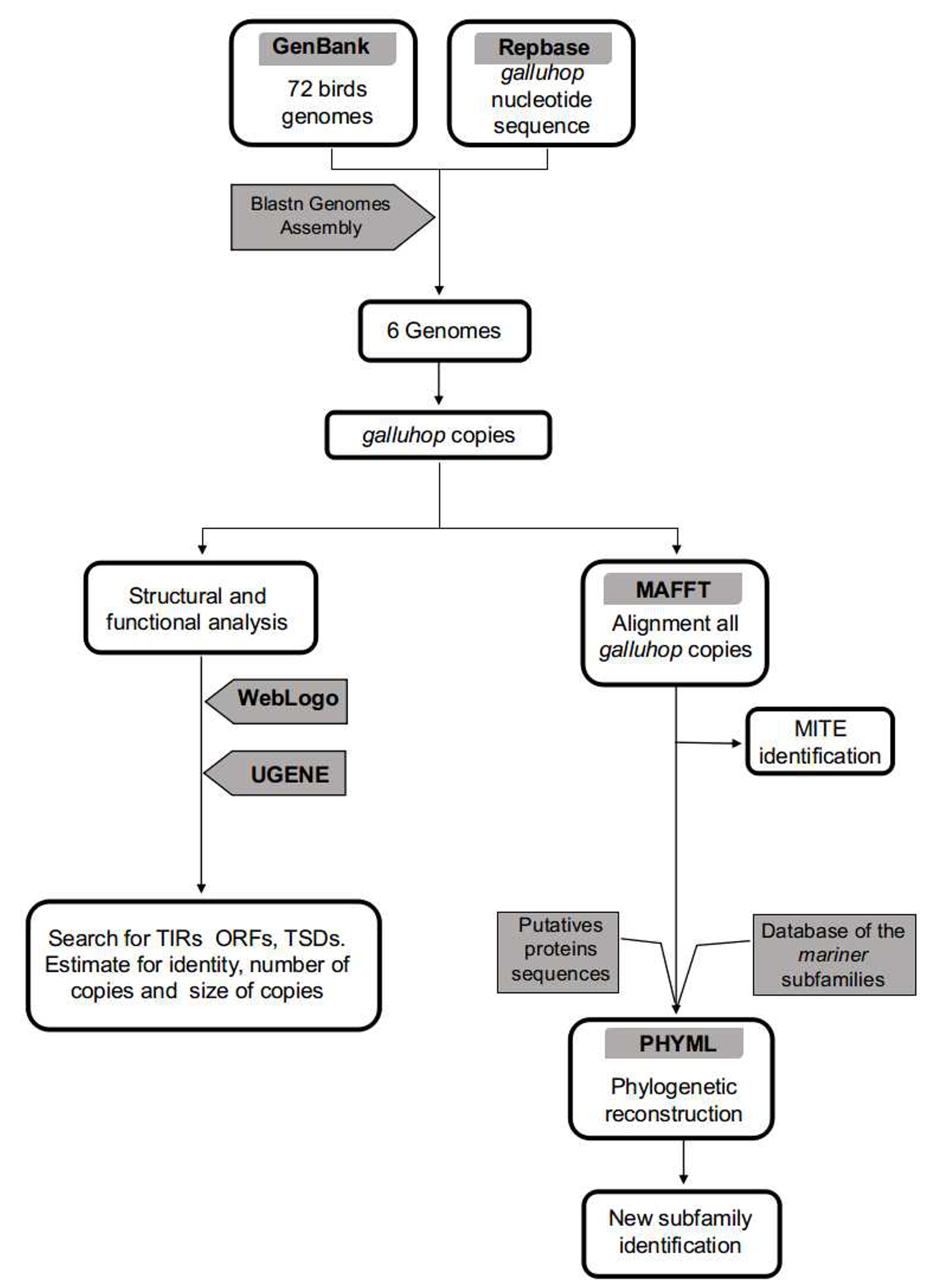

Supplement: Supplementary file 2 — Experimental design procedure showing steps of the analysis. Galluhop homologous sequences were found in 6 of 72 genomes analyzed. We analyzed the functional and structural characteristics and phylogenetic reconstruction of the putative transposases. (TIFF 4512 kb) [file 13100_2017_94_MOESM2_ESM.tif]

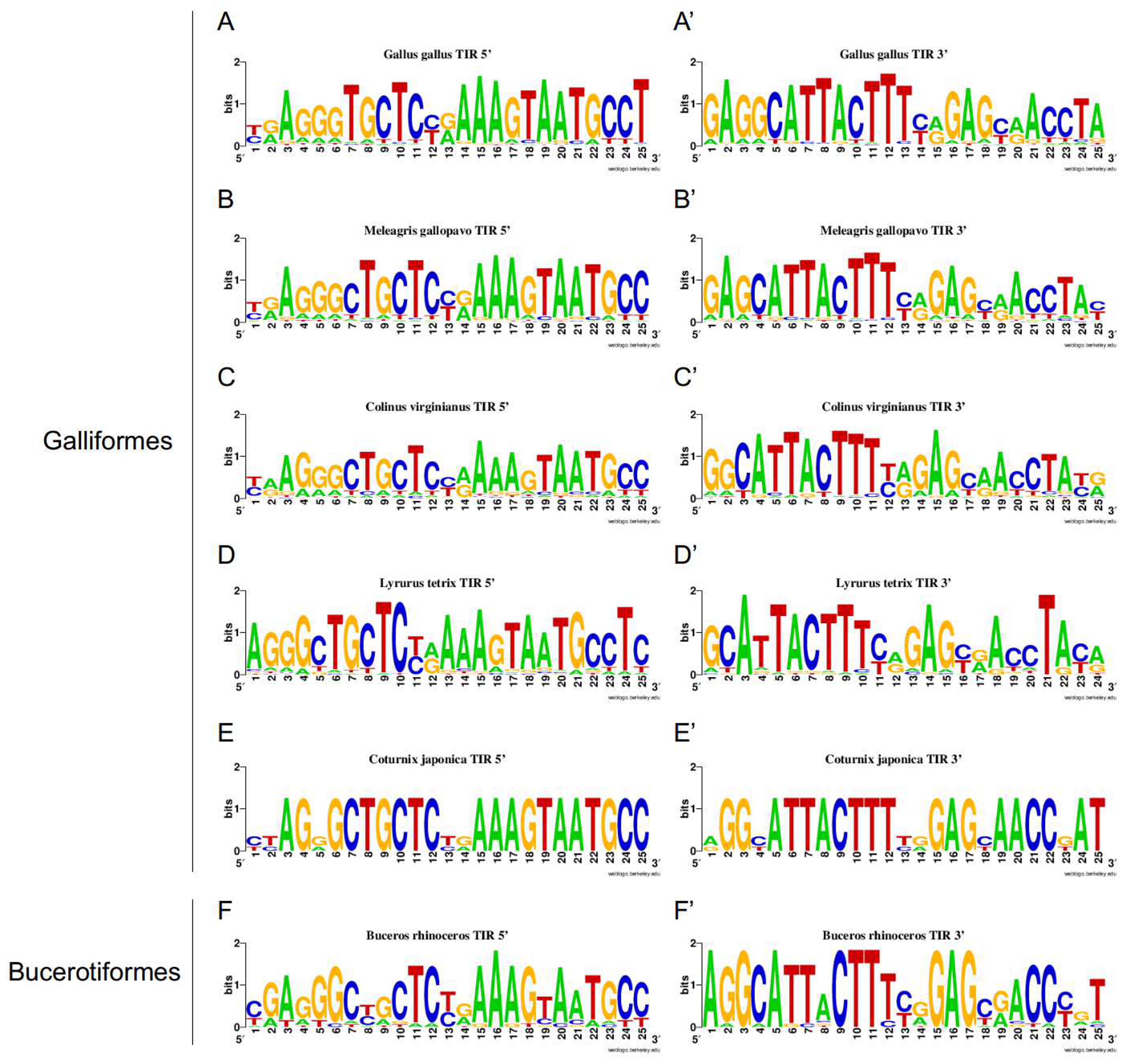

Supplement: Supplementary file 4 — Graphical representation of the conservation of terminal inverted repeats (TIRs). The TIRs 5′ and 3′ galluhop element in the six genomes generated with WebLogo [35]. Order Galliformes: G. gallus (A – A’),M. gallopavo (B – B′), C. virginianus (C – C′), L. tetrix (D – D’) and C. japonica (E – E’). Order Bucerotiformes: B. rhinoceros (F – F′). (TIFF 7747 kb) [file 13100_2017_94_MOESM4_ESM.tif]

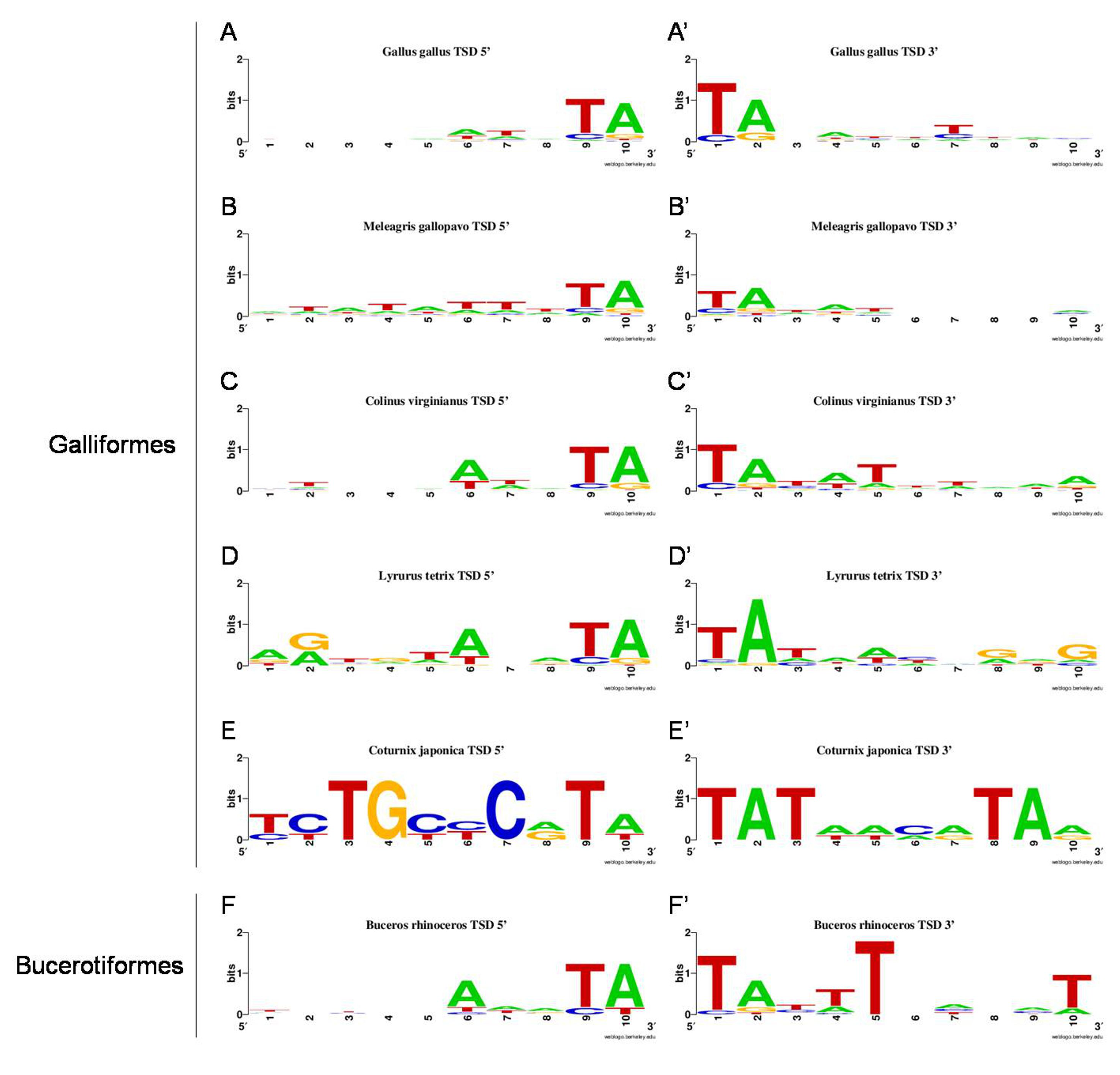

Supplement: Supplementary file 5 — Graphical representation of the conservation of target site duplications (TSDs). The TSDs 5′ and 3′ galluhop element in the six genomes generated with WebLogo [35]. Order Galliformes: G. gallus (A – A’),M. gallopavo (B – B′), C. virginianus (C – C′), L. tetrix (D – D’) and C. japonica (E – E’). Order Bucerotiformes: B. rhinoceros (F– F′). (TIFF 4900 kb) [file 13100_2017_94_MOESM5_ESM.tif]
